# An overview of radiolabeled amino acid tracers in oncologic imaging

**DOI:** 10.3389/fonc.2023.983023

**Published:** 2023-02-17

**Authors:** Sanchay Jain, Vandana Kumar Dhingra

**Affiliations:** ^1^ Department of Nuclear Medicine, All India Institute of Medical Sciences Bhopal, Bhopal, India; ^2^ Department of Nuclear Medicine, All India Institute of Medical Sciences, Rishikesh, India

**Keywords:** radiolabelled, amino acid, PET/CT, imaging, oncology

## Abstract

Molecular imaging has witnessed a great progress in the field of oncology over the past few decades. Radiolabeled amino acid (AA) tracers are particularly helpful in the areas where the utility of 18F-Fluorodeoxyglucose (18F-FDG) positron emission tomography with computed tomography imaging has been limited such as in evaluating brain tumors, neuroendocrine tumors (NETs), and prostate cancer. Radiolabeled AA tracers such as 6-[18F]-L-fluoro-L-3, 4-dihydroxyphenylalanine (18F-FDOPA), 18F-fluoro-ethyl-tyrosine (18F-FET), and 11C-methionine have found wide applications in brain tumors, which, unlike 18F-FDG, concentrate in the tumor tissue to a greater extent than that in normal brain tissue by providing accurate information about tumor volume and boundaries. 18F-FDOPA is also useful in evaluating NETs. Tracers such as 18F-FACBC (Fluciclovine) and anti-1-amino-2-[18F]fluorocyclopentyl-1-carboxylic acid (18F-FACPC) are used in imaging of prostate cancer and provide valuable information of locoregional, recurrent, and metastatic disease. This review highlights AA tracers and their major applications in imaging, *viz*., in evaluating brain tumors, NETs, and prostate cancer.

## Introduction

Cancer is characterized by alterations in cellular metabolism, and these alterations are useful targets for imaging and therapy. 18F-FDG has proved to be a pathbreaking tracer in oncologic imaging and has been widely used in staging, restaging, response evaluation, and prognosticating various cancers. However, there are certain limitations for FDG PET (positron emission tomography) imaging: difficulty in identifying primary and metastatic brain lesions due to high background and low contrast; some cancers such as prostate, NETs, liver cancer, and renal cell carcinoma may show low or non-specific FDG uptake, leading to false-positive or false-negative studies; and low specificity in differentiating inflammation/infection from malignant lesions. Radiolabeled amino acids (AAs) can be used for non-invasive visualization of important metabolic changes and have wide applicability in the oncologic imaging due to biologic diversity of the transport systems of AAs and their metabolic pathways. Certain specific AA transporters play an important role in altered metabolism in various cancers that can be of particular interest for diagnostic and therapeutic applications. AA tracers have been most commonly used for imaging brain tumors, prostate cancer (PCa), and neuroendocrine tumors (NETs) (**Table 1**). However, their applications are widely growing, and promising results have been published from animal studies. This review highlights important tracers, mechanisms, and applications of AAs in oncologic imaging (10, 11).

**Table 1:** Important amino acid tracers.

S. No.	Study (reference)	Agent	Type of cancer
1.	Zhao et al. (1)	11C-MET	Brain tumors
2.	Katsanos et al. (2)	11C-MET and 18F-FET	Brain tumors
3.	Kim et al. (3)	11C-MET	Brain tumors
4.	Dunet et al. (4)	18F-FET	Brain tumors
5.	Galldiks et al. (5)	18F-FET	Brain tumors
6.	Popperl et al. (6)	18F-FET	Brain tumors
7.	Nihashi et al. (7)	18F-FET *vs*. 18F-FDG	Brain tumors
8.	Piccardo et al. (8)	18F-DOPA	NET
9.	Gusman et al. (9)	18F-FACBC (Fluciclovine)	Prostate cancer

## Amino acid metabolism and oncologic imaging: Mechanisms

AAs enter cells through AA transporters, and upregulation of these transporters such as LAT1 and ASCT2 is noted in several cancers, which, in turn, increase uptake of AAs in tumors by several folds. On the basis of the requirement of sodium ions, AA transport system is classified as (i) Na^+^-dependent and (ii) Na^+^-independent transport systems. L-AAs are nutritionally essential for cell growth and maintenance of nitrogen balance, as they play an important role in metabolism, protein synthesis, cellular signalling, gene expression regulation, synthesis of some hormones, neurotransmitters, and other nitrogenous substances. L-AAs are obtained from either intracellular protein recycling or transport from extracellular surroundings *via* transport systems in the cell membrane. System A, which is a member of solute carrier 38 (SCL38) gene family, is a Na^+^-dependent transporter for small aliphatic AAs such as serine, alanine, and glutamine. System L is Na^+^-independent and takes up branched AA such as phenylalanine, isoleucine, tryptophan, valine, methionine, and histidine from the extracellular space. To fulfill their increased demand for proliferation, tumor cells utilize more AAs compared with normal cells. AA transporters have a higher expression in tumor cells, particularly LAT1, ASCT2, xCT, ATB0, etc. ASCT2 and LAT1 reveal a threefold increase expression in most of the cancerous lesions (12–14). Most of the available PET tracers are labeled with positron-emitting radionuclides 18F and C11. 18F-labeled AAs comprise an important and most commonly used class of agents used for oncologic imaging that detect increased metabolism of AAs in the tumor by targeting the upregulated AA transporters. Properties of the ideal labeled AA tracers include a simple and practical labeling technique, quick transport to tumor cells, high uptake and optimum retention time, no affinity toward inflammatory tissue components, high plasma clearance, and good Blood brain barrier (BBB) permeability (when used for brain tumor evaluation) (15–17).

## Brain tumors

Neuroimaging plays a crucial role in the management of primary and metastatic brain tumor. Gliomas are the most common intra-axial brain tumors in adults and are graded from I to IV. High-grade gliomas (III and IV) are aggressive and treated with combinations of surgery, radiotherapy, and chemotherapy. Low-grade gliomas are slowly growing and carry comparatively better long-term survival but may undergo transformation into high-grade gliomas. Grade, extent, and location of gliomas are key characteristics in planning treatment. Radiolabeled AA tracers can be advantageous over 18F-FDG PET/computed tomography (CT) by helping in better delineation of tumor boundaries, targeting biopsy sites, and radiotherapy. Most commonly used AA tracers used in evaluating brain tumors are 11C-MET (methionine), 18F-FET, and 18F-FDOPA. Contrast-enhanced MRI plays a key role in evaluating gliomas that includes diagnosis, staging, treatment response evaluation, and assessment of recurrence but cannot accurately evaluate non-enhancing parts of gliomas, as some part of the glioma may have relatively intact BBB and do not show contrast enhancement. It is also difficult to distinguish whether a recurrence or residual tumor from radiation necrosis PET imaging with 18F-labeled AAs can delineate metabolic tumor volume (MTV) and tumor metabolic load and whether it is more useful in treatment response evaluation than MRI. Compared with 18F-FDG PET/CT, 18F-labeled AAs offer an advantage of a low uptake and retention in the normal brain and their ability to delineate the entire tumor volume. 18F-FDOPA, [18F]6-fluoro-3-O-methyl-L-3,4-dihydroxyphenylalanine (18F-OMFD) (metabolite of 18F-FDOPA), 18F-FET, L-[3-(18)F]-α-methyltyrosine (18F-FAMT), 2-18F-fluoro-L-tyrosine (2-FTyr), 18F-BPA (boron phenylalanine), (4S)-4-(3-[18F]fluoropropyl)-L-glutamate (18F-FSPG), and 4-18F-(2S,4R)-fluoro-glutamine (18F-FGln) are useful PET radiotracer for evaluating gliomas of which 18F-DOPA (derivative of L-tyrosine) and 18F-FET (derivative of L-phenylalanine) are most commonly used. 18F-FET PET/CT provides a good contrast and is a valuable modality for diagnosing primary brain tumors, distinguishing low-grade from high-grade gliomas, and distinguishing recurrent brain metastasis from radiation necrosis after radiotherapy, and offers an added advantage in cases of brainstem and spinal cord gliomas when compared with MRI. 18F-FET shows a lower uptake by inflammatory cells than 11C-MET or 18F-FDG, thereby clearly delineating tumor from inflammation (18, 19).

11C-MET has a superior diagnostic accuracy than 18F-FDG in detecting, grading, and delineating boundaries; detecting recurrences; prognosis prediction; and treatment response evaluation in higher-grade gliomas (19). In the meta-analysis by Zhao et al., the pooled sensitivity, pooled specificity, and area under the receiver operating characteristic curve (AUC) for differentiating brain tumors of 11C-MET PET were 0.91, 0.86, and 0.94, respectively, whereas that of 18F-FDG were 0.71, 0.77, and 0.80, respectively. These findings of this meta-analysis suggest that 11C-MET PET has excellent diagnostic accuracy in differentiating brain tumors. Moreover, 18F-FDG-PET has a limited role in brain tumor differentiation (1). In another meta-analysis by Katsanos et al., the pooled sensitivity rates of MET PET (0.94) and FET PET (0.88) were found to be higher than that of FDG PET (0.63) (P < 0.001). However, the pooled specificity of FDG PET (0.89) was higher than that of MET PET (0.55) and FET PET (0.57) (P = 0.002). In addition, this meta-analysis showed the superiority of FDG PET due to higher positive likelihood ratio than FET PET and MET PET (2). The meta-analysis by Kim et al. concluded that the tumor-to–normal tissue ratio (TNR) and MTV of 11C-MET PET are significant prognostic parameters in gliomas. Higher TNR poses higher risk of death, and higher MTV has a higher risk of adverse events or death. The effect of the TNR and MTV on survival was determined by the effect size of the hazard ratio (3).

In the meta-analysis by Dunet et al., 18F-FET PET has a pooled sensitivity of 0.82, specificity of 0.76, and AUC of 0.84 for the diagnosis of primary brain tumors and concluded that 18F-FET PET has excellent performance for diagnosing primary brain tumors (4). In the study by Galldiks et al., 18F-FET PET was found to have a high accuracy in differentiating local brain metastasis recurrence from radionecrosis, as assessed by mean and maximum tumor-to-background ratios (TBRs) that were significantly higher in recurrent metastasis than that in radiation necrosis (P < 0.001) (5). Popperl et al. evaluated the potential of FET PET for tumor grading in untreated patients. The results of their study show significant difference (P = 0.001) between low-grade and high-grade gliomas using the standard method by calculating TBR. In addition, a dynamic evaluation distinguished low-grade gliomas from high-grade gliomas with a higher diagnostic accuracy (sensitivity of 0.94, specificity of 1, and AUC of 0.967) (6).

Nihashi et al. studied the diagnostic accuracy of PET imaging for diagnosis of recurrent glioma. The results of their meta-analysis showed a pooled sensitivity of 0.70 for 11C-MET PET and 0.77 for FDG PET. The pooled specificity was found to be 0.93 for 11C-MET PET and 0.78 for 18F-FDG PET. They concluded that 18F-FDG and 11C-MET PET have a moderately good accuracy for diagnosing recurrence in gliomas suspected by CT or MRI (7).

18F-FDOPA PET/CT is useful in detection of primary, metastatic, and recurrent brain tumor, delineating tumor volume as well as determining and grading proliferative activities. Visual and semi-quantitative indices in 18F-DOPA PET provide a high accuracy for detection of recurrence in glioblastoma and predict progressive-free survival, and 18F-DOPA PET/CT or PET/MRI can detect a striatal involvement in pediatric gliomas. 18F-BPA is useful in the boron neutron capture therapy of gliomas and other head and neck cancers, as it is used for the TNR. 18F-Gln demonstrates a high tumor uptake in the gliomas but a low background brain uptake, which helps in clear delineation of tumor (18, 19).

## Neuroendocrine tumors

NETs originate from peptidergic neurons and neuroendocrine cells and originate in different locations. Clinical presentation and symptoms occur depending upon the biologically active substances such as hormones or vasoactive peptides. Common NETs include carcinoid tumors, pheochromocytoma, medullary thyroid cancer, small cell lung cancer, and neuroblastoma (20). 18F-FDG is a non-specific tracer for evaluating NETs, and 18F-DOPA PET/CT is sensitive and comparatively better than CT or MRI in posttreatment evaluation of pheochromocytoma and paraganglioma. 18F-DOPA is also useful in evaluating Gastroenteropancreatic neuroendocrine tumors (GEPNETs) and neuroblastomas. 18F-DOPA PET/CT is more sensitive than CT and SRS in detecting positive lesions of carcinoid tumors. However, obviously, the somatostatin receptor expression cannot be evaluated using 18F-DOPA PET/CT. 18F-DOPA is also useful in the evaluating MTC. NETs have increased activity of LDOPA decarboxylase and therefore show a high concentration of 18F-DOPA. 18F-FDOPA PET is more sensitive than 11C-HTP in gastrointestinal NETs. For pancreatic NETs, the results are opposite (21–23).

In the systematic review and meta-analysis for head-to-head comparison between 18F-DOPA and 68Ga-DOTA peptides PET/CT in detection of intestinal NETs by Piccardo et al., they calculated sensitivity in terms of patient-based analysis (PBA), region-based analysis (RBA), and lesion-based analysis (LBA). A non-negligible difference in favor of 18F-DOPA PET/CT was found (95% *vs*. 82%) on LBA, i.e., lesion detection. However, no significant difference was observed in PBA and RBA (8).

Lee et al. conducted a network meta-analysis by comparing five different PET tracers for detecting medullary thyroid carcinoma. They compared 18F-FDG, 18F-DOPA, 68Ga somatostatin analogs, 18F-FDOPA, and 11C-MET. 18F-DOPA PET had a significantly higher detection rate than FDG PET in PBA and LBA (OR of 2.44 and 5.74, respectively). In addition, regardless of serum calcitonin or Carcinoembryonic Antigean (CEA) levels and calcitonin doubling time, 18F-DOPA showed the highest surface under the cumulative ranking curve value in both PBA and LBA (24).

## Prostate cancer

PCa is complex and biologically heterogeneous tumor, which is one of the most common malignancies in men. Accuracy of CT and MRI is limited in detecting primary tumor and metastases in regional lymph nodes. Overexpression of several AA transporter systems is noted in PCa, particularly the system ASC transporter ASCT2 and the system L transporter LAT1, which are associated with tumor aggressiveness and poor survival. Fluciclovine is not metabolized and is not incorporated in proteins (25, 26).

FDG uptake is lower in PCa lesions. 18F-FACBC (fluciclovine) (L-leucine analog) and 18F-FACPC (analog of FACBC) are labeled AA tracers, which provide higher sensitivity and specificity rates in detecting tumor and metastatic lymph nodes. The pathologic activity of 18F-FACBC in prostate tumors and nodal metastatic disease peaks rapidly (4–10 min) due rapid influx and efflux of AAs. Another advantage of 18F-FACBC is its slow excretion into the bladder that is helpful in clear visualization of adjacent pelvic lesions. 18F-FACBC PET/CT also helps to assess aggressiveness of tumors by differentiating between cancer tissue and benign tissue. 18F-FACBC is transported into cells by LAT1 and ASCT2. 18F-FACBC appears to be superior to choline in biochemical relapse and is approved by the United States Food and Drug Administration (US FDA) for the detection of recurrent PCa (9, 27). 18F-FACBC is also superior to 111In-capromab and 11C-choline and also offers an advantage of a longer half-life compared with C11-choline (28).

18F-FACBC offers practical advantages in terms of biodistribution (negligible urinary activity in one hours after injection), image quality, lower background, and acquisition protocol that improves lesion detection. Although 18F-FACBC seems to be highly sensitive for detecting recurrent disease but has low-to-moderate specificity at its best, with a relatively higher rate of false positivity that may falsely upstage the disease. Studies comparing 11C/18F-choline with 18F-FACBC are few. In general, 18F-FACBC is superior in detecting biochemical recurrence and outperforms 11C-choline in detecting true-positive and true-negative lesions in prostate bed, lymph nodes, and bones (27, 29, 30).

## Other tumors

Sensitivity of 18F-FAMT is shown to be higher than that of 18FFDG in evaluating maxillofacial tumors. 11CMeAIB can be used in imaging of head and neck cancers and also in pulmonary and mediastinal mass lesions. 18F-DMT (tyrosine derivative) that is transported *via* LAT1 has shown a higher specificity than 18F-DFMT in Non-small cell lung cancers (NSCLC) and head and neck cancers. 18F-FBPA (4-borono-2-18F-fluoro-phenylalanine) predicts 10B concentrations after administration of boron-containing drugs for neutron capture therapy. 18F-FACBC PET/CT can be used in visualization of invasive lobular and invasive ductal breast cancers, and 18F-FACBC uptake is significantly higher in primary and metastatic lesions of breast cancer such as in lymph nodes and in bones than that in benign or normal breast tissues. However, liver metastasis could not be sensitively detected because of a high background physiological uptake. On treatment response evaluation, changes in 18F-FACBC avidity correlated well with a percentage tumor reduction caused by treatment. 18F-5-fluoro-aminosuberic acid (18F-FASu) has been tried for diagnosis and treatment response evaluation in certain breast cancers. 18F-FGln can be used to track the glutamine pool size of the cell in triple-negative breast cancer (31–36).

## Conclusions

Although AA tracers do not have as widespread applications as 18F-FDG, they have certain specific and valuable applications in oncologic imaging especially when they overcome the shortcomings of 18F-FDG PET/CT imaging. 18F-FDOPA and 18FFET have a well-established role in diagnosis, staging, treatment response, and evaluation of recurrence in gliomas. In addition, 18F-FDOPA is also useful in assessment of NETs where it has shown a high sensitivity in evaluating posttreatment lesions of pheochromocytomas and paragangliomas. It is also useful in imaging GEPNETs and neuroblastoma. 18F-FACBC and 18F-FACPC can accurately detect primary tumor and regional metastatic lymph nodes in PCa. However, the role of radiolabeled AA is limited to diagnostic imaging only; as with the evolution of theranostics, other tracers have a larger role in imaging and treatment of NETs and PCas. Glutaminolysis is upregulated in several cancer cells, which provides alternate source of carbon and energy; therefore, glutamine is an area of interest. Radiolabeled glutamine analogs would allow to explore this arena in human cancers. Transporter system L has been a key area for developing tracers for tumor imaging (e.g., 11C-MET, 18F-FET, and 18F-FDOPA), which has also resulted in several radiolabeled AA tracers that are effective for imaging NETs (18F-FDOPA) and PCa (18F-FACBC). Although AA-based imaging has evolved over the years, some challenges need to be addressed for further expansion of their applications such as a better understanding of their transport mechanisms, deeper insights into structure–activity relationships for tracers designing for specific transporters, and clinical application of these tracers into current grey areas in oncologic imaging.

## Author contributions

Dr SJ- Preparation of manuscript. Dr VD - Conceptualising, edits and finalised version. All authors contributed to the article and approved the submitted version.
